# Longitudinal Evaluation of Transition Services (“LETS Study”): Protocol for outcome evaluation

**DOI:** 10.1186/1471-2431-12-51

**Published:** 2012-05-15

**Authors:** Irina Tsybina, Shauna Kingsnorth, Joanne Maxwell, Mark Bayley, Sally Lindsay, Patricia McKeever, Angela Colantonio, Yani Hamdani, Helen Healy, Colin Macarthur

**Affiliations:** 1 Bloorview Research Institute, Toronto, Canada; 2 Holland Bloorview Kids Rehabilitation Hospital, Toronto, Canada; 3 Toronto Rehabilitation Institute, Toronto, Canada; 4 Dalla Lana School of Public Health, University of Toronto, Toronto, Canada; 5 SickKids Research Institute, Toronto, Canada

**Keywords:** Transitions, Youth, Complex chronic conditions, Disability, Rehabilitation, Study protocol, Research methods

## Abstract

**Background:**

Because of advances in medical treatment, most children with physical disabilities can expect to achieve near normal life spans. Typically, coordinated teams of health care providers in specialized pediatric settings care for these children. As these children reach adulthood, however, the availability of services and expertise changes because the adult health care system has different processes designed to meet their specialized needs. Gaps in continuity of care during the transition from pediatric to adult services, and associated poor health outcomes are well documented. In response, new models of care are being introduced to address the complex process of health care transition. This paper describes a study protocol of a client-centred, prospective, longitudinal, mixed-method evaluation of linked model of health care across the lifespan (the LIFEspan Model), offered by a pediatric rehabilitation centre and an adult rehabilitation centre.

**Method:**

This project will include a process and an outcome evaluation of the LIFEspan Model. The *process evaluation* will detail the specific service delivery that occurs with respect to preparation for transition and transfer of care through chart audits of pediatric medical records and qualitative interviews with LIFEspan staff. The *outcome evaluation* will measure the effect of the model on: 1) maintaining continuity within the health care system from pediatric to adult care; and 2) secondary outcomes related to health, well-being, social participation, transition readiness, and health care utilization of youth with cerebral palsy and acquired brain injury. Standardized instruments will include Health Utilities Inventory, Assessment of Life Habits, Arc’s Self-Determination, Assessment of Health-Related Quality of Life, Partners in Health Questionnaire, Social Support Questionnaire, and Self-Efficacy for Managing Chronic Disease.

**Discussion:**

The LETS study will be original in its undertaking of a prospective examination of outcomes 1-year post-transition, use of multiple comparison groups, and absence of disability-related exclusion criteria ensuring that the transition experiences of varied populations of young people and their families will be represented.

**Trial registration:**

http://www.clinicaltrials.gov, ID NCT00975338

## Background

Effective transition of adolescents from pediatric to adult health services has become a prominent issue in health care research. As a result of unprecedented advances in medical practice over the past three decades, as many as 50 - 90% of children with congenital or acquired physical disabilities now reach adolescence and live into adulthood [1-6]. For example, in 1995, twice as many newborns with spina bifida survived in the US as compared to 1975 [3]. Consequently, a new cohort of adults with childhood-onset disabilities and complex chronic conditions has emerged, requiring appropriate transitional care upon their “discharge” from pediatric facilities.

Transition from the pediatric to adult health care system is a complex process that must be addressed in a holistic manner inclusive of medical, psychosocial, educational, and vocational components [7,8]. Ideally, health care during the period of transition should be delivered in a coordinated and uninterrupted manner through the provision of developmentally appropriate and comprehensive services [7]. However, finding appropriate adult care is challenging [9], as many adult programs have eligibility criteria that focus on adults with new onset disability issues [10], and there is a general lack of expertise among most adult health care providers related to aging with a childhood-onset disability [4,11,12]. In the absence of an adequate system of care, adolescents with disabilities are often significantly under-serviced as young adults, and many receive no care whatsoever [6,13,14].

Even when links to adult care are established, the process of transition remains difficult. First, there are important differences between the pediatric and adult health care environments, such as decreased family involvement and highly specialized, fragmented care that characterize the adult health care system [15-18]. To succeed in this system, young people need to learn how to manage their own health [19]. Unfortunately, the pediatric system has not emphasized developing the skills of children with disabilities to enable them to navigate the adult system that requires more self-advocacy and self-determination skills than the pediatric sector, where parents often play this role [20,21]. Second, adolescence is a critical developmental phase between childhood and adulthood, characterized by increased socio-cultural turbulence and vulnerability for all adolescents, regardless of disability [19,22]. Two of its hallmarks are a search for an identity separate from that of the family of origin and re-defining relationships with adults in parental and caring roles [23]. These may contribute to tension in the relationships of adolescents with their families and health care providers.

This difficulty in establishing continuity of care from the pediatric to adult system and inadequate transition preparation often results in unfavourable outcomes for young adults with disabilities. Gaps in transition to the adult health system result in poor health outcomes and diminished opportunities to participate as productive members of the community [6,24]. Lack of continuity is especially detrimental to populations with demanding and complex health care needs. For example, Young et al. [14] reported a significant drop in self-reported health status from youth to adulthood in young people with acquired brain injury (ABI), cerebral palsy (CP), and spina bifida (SB). In the absence of community and primary care services, health issues go unmonitored, putting these individuals at further risk for developing preventable secondary complications [25]. This paradoxically has led to increased utilization of health services (e.g., inpatient hospitalizations) and inappropriate reliance on emergency health services (e.g., use of walk-in clinics and emergency departments) [10,14,26].

Despite much pressure to identify best practices in transition, research has only recently started to move from consensus reports to formal evaluations. The research to-date has focused largely on the need for transitional care [19] and articulating challenges to implementing effective models of care [9,10,13,17]. Empirical evidence addressing processes and outcomes of transition remains limited [27,28]. The majority of recent papers on transition focus on generating recommendations for supporting transitions, while few qualitative studies and one prospective evaluation provided empirical data [29]. To our knowledge, only seven studies described in the transition literature on physical disability (SB [29]) and chronic illness (type 1 diabetes, cystic fibrosis, juvenile arthritis, epilepsy [30-35]) were formal evaluations of transition care that included prospective data collection. Of these, no studies were longitudinal or followed the participants significantly past the transitional period, and three studies were qualitative and thus lacked a comparison group and dealt with patient perceptions rather than provided measures of transition outcomes [31,32,34]. Only four studies [29,30,33,35] examined health status, health care utilization, quality of life or drop-out rates using objective measures. It is difficult to draw conclusions from these studies because of the variability in the sample sizes, diagnoses, measures, types of comparison groups, and international differences in models of health care provision affecting access to services (e.g., health care insurance differences in the US and Canada). In addition, participants in the majority of these studies had a chronic illness as their primary diagnosis [30-35], rather than a physical disability. Although these chronic conditions can be quite physically disabling, transition preparation for these youth may differ from that for youth with disabilities in having a much more specific self-management focus. Thus, research in transition care for these clients is rooted in adult literature that does not take into account the shift of responsibility from parent to youth [36,37]. Finally, the optimal window for measuring continuity of care is not yet known, and cross-sectional studies might not have reflected the outcomes of youth who ‘fall through the cracks in the system’ several years after discharge from the pediatric system. In summary, the current status of childhood disability transition literature is characterized by methodological limitations, lack of definitive results or focus on physical childhood-onset disability, and absence of mixed-method longitudinal studies that would capture both objective measures of functional status and patient/clinician experiences [19].

### The “LETS Study”

The “Longitudinal Evaluation of Transition Services” (“LETS Study”) study will attempt to address the many gaps identified in the literature by conducting a formal, prospective, longitudinal, mixed-method evaluation of the LIFEspan (“Living Independently and Fully Engaged”) model of transition care [38]. LIFEspan is a recently funded, coordinated, client-centred model of linked health care across the lifespan, offered through a partnership between a pediatric rehabilitation centre and an adult rehabilitation centre. The study will compare adolescents with the diagnoses of CP and those with ABI, who receive LIFEspan, to a group of adolescents with SB who will not receive LIFEspan. A second cohort of young adults with CP and ABI who had been discharged prior to the formal launch of LIFEspan, will also be used as a comparison group. The findings of this work will inform the understanding of delivery of transitional care services for young people with childhood-onset disabilities. The objective of this paper is to serve as the first step in knowledge dissemination related to the LETS study. The current paper outlines the study protocol, explains the rationale for the study design and selection of outcome measures, and documents several methodological challenges encountered.

### Hypotheses

The primary quantitative outcome of the study will be participants’ post-transition continuity of care, as defined in the “Measures” section of the protocol. We hypothesize that continuity within the health care system after transitioning from pediatric to adult care will be maintained by those participants who receive LIFEspan. Secondary outcomes of interest will include health and well-being, activities and social participation, transition readiness, and health care utilization (frequency, type and duration of emergency services and hospitalizations). It is further hypothesized that youth who will have experienced continuity of care will have enhanced access to health care professionals, reduced emergency health care use, better health status and well-being, more involvement in the community, and greater self-determination, self-efficacy, and self-management skills, as compared with youth not having received LIFEspan. In-depth qualitative analyses of interviews conducted with parents and youth will further inform our understanding of the transition experience. In addition, a detailed chart audit will provide a comprehensive description of the services received by participants, in order to ascertain the model’s treatment fidelity.

## Methods

### Intervention

The LIFEspan model involves a unique partnership between a pediatric and an adult rehabilitation centre to offer a continuous model of care. The LIFEspan model attempts to address the need for continuity of care by engaging adolescents with childhood-onset disabilities in a two-year transition preparation, then coordinating the transfer of their care through a formal linkage and cross-appointed health care providers between the two academic pediatric and adult health sciences centres. The model is described in detail by Kingsnorth et al. [38].

### Design

To bridge the pediatric-adult divide, health care providers from both systems were engaged in the development of this project as part of the research team. The study will consist of process and outcome components. The process evaluation will detail the specific service delivery that occurs with respect to LIFEspan preparation for transition and transfer of care, and will describe the pediatric clinical interventions and processes of the two year preparation period. It will include a comprehensive chart audit and interviews with LIFEspan staff. Findings from Phase 1 of these interviews (i.e., enablers and barriers to the development and implementation of the LIFEspan model) have been reported [38]. The emphasis of the current paper will be to outline the outcome evaluation component.

### Ethics

The study has been granted ethics approval at each of the partnering hospitals and institutions providing the data sources: Holland Bloorview Research Ethics Board (Approval Number: 09–035) on August 17 2009, Toronto Rehabilitation Institute Research Ethics Board (Approval Number: 10–009) on May 10 2011 and Sunnybrook Health Sciences Centre Research Ethics Office (Approval Number: 251–2011) on September 27 2011. This study has been registered as a clinical trial ( http://www.clinicaltrials.gov, ID NCT00975338).

### Participants

Currently, the LIFEspan model is only available to current pediatric clients with a diagnosis of CP or ABI. Thus, the ideal comparison group would be age- and disability-matched clients who do not receive LIFEspan; however, all clients with these conditions are streamed into this service according to institutional policy. Random assignment of ABI and CP clients to a control condition was considered unethical given our understanding of the poor outcomes associated with inadequate preparation for transition and the existing gaps in adult health care services [11,12]. Furthermore, the service is meeting client demand; therefore, no waiting list for services has been developed, eliminating this alternative as a comparison group option. Recruitment at multiple sites would also introduce further heterogeneity into the sample due to the lack of standardization in transition practices across agencies. Therefore, ethically, the best comparison group to support collection of the primary and secondary outcomes of interest is that of current pediatric clients with SB, as they face similar challenges with respect to complexity of care, the need for ongoing monitoring, and holistic support to maximize their social participation and community involvement. Further, young people with SB experience the same gaps in obtaining adult health care services and demonstrate comparable health and participation related outcomes as a result [13,14,39]. Despite this strategy, there is the potential that the inclusion of SB participants may confound the results because of differences in medical issues. Therefore, the study will also include a retrospective component, to allow for comparisons of continuity of care in the year following discharge for clients with the same diagnosis (ABI or CP) but who received different interventions; that is, LIFEspan vs. ‘standard of care’, prior to the introduction of LIFEspan, which would typically involve discharge without transition preparation.

The intervention group will consist of 30 youth with ABI and 50 youth with CP enrolled in LIFEspan. The two comparison groups will consist of youth not enrolled in this service model: 21 youth with SB (prospective cohort) and 15 young adults with ABI and 20 with CP (retrospective cohort). Data from each participant group will be collected from a matched window of time, corresponding to the participants’ 16^th^ to 19^th^ birthday. All parents of participants from the prospective groups will be eligible to participate as secondary participants in a qualitative component of the outcome evaluation. Recruitment for this component will be built into recruitment of the primary study participants.

#### Proxy reporting

Given the disability groups included in this study, a tremendous variation in the range of participants’ functional, communicative and cognitive abilities is expected. Varied strategies for enhancing participant autonomy and supporting communicative abilities will be implemented, such as proxy reporting and special accommodations. Ethical considerations in participants’ abilities to consent to research participation will be addressed as described below. However, data will be coded as self-report or proxy to explore potential patterns as they might emerge.

#### Consent

A study information letter will be sent to all eligible families, followed by a phone call. Informed consent will be obtained from all eligible participants who express interest in participating, and if required, from substitute decision makers.

The study will be introduced to all prospective participants in an in-person meeting, where the study personnel will use her judgment to determine whether the youth would provide consent or assent. If a participant demonstrates a clear understanding and appreciation of the purpose of the study and his/her rights regarding participation, consent will be obtained directly. If the young person demonstrates a partial understanding, parental consent and participant assent will be obtained. If a participant does not have a reliable means of communicating yes/no, consent for study participation will be obtained from a substitute decision maker, such as a parent. Prospective youth consent will include acknowledgement of the quantitative assessments of secondary outcomes related to health, well-being, social participation, and transition readiness; permission for the research team to access their health care utilization data held within the administrative databases maintained by the Ministry of Health and Long-Term Care (MOHLTC) and individually identified primary pediatric and adult health care facilities, and participation in qualitative interviews regarding their transition experience.

All retrospective participants will receive the consent packages by mail if they express interest in participating during the initial phone call. Thus, the parent and youth will be responsible for making this decision jointly, and they will be sent all versions of the consent materials. Retrospective primary participant consent will include the release of health care numbers and permission to review pediatric and adult health service use through identified administrative database reviews and chart audits. All participants will be informed at the time of consent that they can withdraw from the study at any point in time. Data collection would cease as of the date of withdrawal and outstanding MOHLTC information release forms would be destroyed.

### Measures

#### Quantitative data

The identification of specific measures for the study was influenced by the transition literature [13,14]; however, the availability of outcome measures straddling the significant developmental periods arising between infancy and adolescence with consideration of varied cognitive impairments and normative trajectories has long posed a challenge in pediatric rehabilitation [40]. Beyond measures of quantity and quality of care (e.g., satisfaction with service delivery, medical record documentation) and continuity of care (e.g., attendance rates, patient satisfaction, treatment adherence, etc.), there are no universally identified outcomes that define a ‘successful’ transition [8,30,34,41,42], as the complexity of interaction of contributing factors is not well understood [8,43]. In this study, the International Classification of Functioning, Disability and Health (ICF) will be used to ensure a broad focus on body function and structure, activity, and participation [44]. The LETS study will examine several groups of outcomes of the transition process as facilitated by the LIFEspan model. Thus, we will use a combination of measures that assess skills that have been identified in the literature through descriptive research as central to successful transition (although yet not empirically proven). In doing so, the choice of study measures reflects a multidimensional approach to transition. The outcomes of the LETS study and corresponding measures for data collection related to each outcome are summarized in Table 1. 

**Table 1:** Outcomes and Measures

**Outcomes**			**Contributors**	**Quantitative Data Sources**		
				**MOHLTC**	**Chart**	**Self-Report**
**Primary**	Pediatric & Adult Medical Specialists		All	☑	☑	
	Family Doctor		All	☑		
	Pediatric Allied Specialists		All		☑	
	Adult Allied Specialists		Prospective			☑ Allied Care Form (LETS Study-Specific Questionnaire)
**Secondary**	Emergency Care & Hospitalizations		All	☑		
	Transition Readiness		Prospective			☑ *Self-Management:* Partners in Health Scale [45]
						☑ *Self-Determination:* Arc’s Self-Determination Scale [46]
						☑ *Self-Efficacy:* Self-Efficacy for Managing Chronic Disease [47]
	Participation		Prospective			☑ *Participation:* Community Involvement Form (LETS Study-Specific Questionnaire)
						☑ *Life Habits:* “Assessment of Life-Habits” [48]
						☑ *Social Support:* Multidimensional Scale of Perceived Social Support [49]
	Health & Well-Being		Prospective			☑ *Health Status:* Self-Rated Health Scale [50]
						☑ *Quality of Life*: Assessment of Health-related Quality of Life [51], Health Utilities Index [52]
**Demographics**			All		☑	☑ Demographic Information Form (LETS Study-Specific Questionnaire

For the primary outcome of interest related to the LIFEspan model of linked health care, we will capture continuity of care between the ages of 18 (discharge from the pediatric centre) and 19 years (one year post-discharge). Continuity of care is not an all-or-nothing outcome [44]; therefore, a study-specific scale will be used to classify participants as having continuity of care on several levels (i.e., achieving a formal discharge from a pediatric facility; having a referral or seeing at least one medical specialist analogous to currently seen pediatric professional; having access to a family physician, etc.). The continuity of care score will be assigned to each participant in their final year of this longitudinal study based on reviewing their health care utilization data, which will include primary care as reflected by MOHLTC summaries, and transition care as abstracted from participant charts (Table 1).

Secondary outcome measures will include patterns of hospitalizations and emergency care utilization, and measures of health and well-being, participation, and transition readiness. As summarized in Table 1, patterns of hospitalizations and emergency care will be determined from MOHLTC summaries that provide information on frequency and duration of access, which we expect will vary as a function of continuity of care. All other outcomes will be obtained through personal assessments using standardized and study-specific tools. A demographic questionnaire and community participation form were developed specifically for the study. Three different standardized measures of health and well-being will be used: the self-rated health scale from the National Health Interview Survey [50], the Health Utilities Index (HUI3 [52]), and the Assessment of Health-related Quality of Life (AQoL [51]). Young et al.’s work provides health status scores for the HUI3 and the health scale for youth and adults with CP, ABI, and SB [13]. These historical data will be valuable in understanding the patterns of change in the current study. The LIFE-H [48] and a survey on community involvement, developed for the study, will be used to assess social participation. However, currently there are no measures available regarding youths’ transition readiness; that is, their capacity to navigate the adult system as a function of transition preparation. Thus, to provide an approximation of this capacity, Arc’s Measure of Self-Determination (capacity to determine their own fate) [46], Self-Efficacy for Managing Chronic Disease (capability to perform in a goal-directed manner) [53], and Partners in Health (PIH) self-management questionnaire (the ability to participate in the management of one’s health condition) [45] will be used. All standardized measures selected for the study have good psychometric properties [45,46,51-57].

Finally, a chart audit tool will be used to abstract information from medical records and to comprise a master record for all visits made during the identified timeframe for each clinical group. The audit tool will include basic demographic data such as date of birth, gender, disability diagnosis, severity, and level of functioning (e.g., Gross-Motor Function Classification System). Cognitive level and educational status, communicative ability, ambulation status, and living arrangements will also be noted if information had been documented in the chart. An additional demographic questionnaire will also be completed by the participants and capture similar information. With respect to service delivery, data including visit dates, discussion of key domains, health care professionals seen and referrals made, discharge summary, date of discharge/transfer, name of primary care provider, and name of follow-up provider will be collected. This information will provide a comprehensive description of the transition services received by the participants as part of the process evaluation, and will serve to explain the trends that emerge in the outcome evaluation.

#### Qualitative data

To further understanding of the patterns of primary and secondary outcome data, participants (if feasible) and their caregivers will be asked to participate in a 45 minute in-depth interview as part of the 1 year post-transfer data collection period. In addition to exploring changes in health and social services, guided, semi-structured questions regarding the transition experience (e.g., experience with continuity of care, preparation for transfer, health, social participation, and their recommendations) will be posed. Such an approach will help foster flexibility and is particularly well suited to exploring the transition process. Although this is a semi-structured format, participants will be encouraged to talk freely about their experiences.

### Data collection

#### Study timeline

The inclusion of three different disability groups raises the possibility of differences arising as a function of disability-related influences rather than the transition intervention. To offset this possibility, baseline measures will be sampled twice to document existing differences within the three prospective participant groups and reduce individual variability, resulting in three different data collection periods for ABI and CP groups (Table 2). The first baseline will occur at 17 years of age and correspond to the completion of year 1 of LIFEspan. The second will occur at 18 years of age, just prior to discharge, corresponding to the end of year 2 of LIFEspan. Follow-up measurement of outcomes of interest will occur at 19 years of age and correspond to 1 year post-transfer. Additionally, participants will be contacted bi-annually to complete specific survey materials (i.e., community involvement and allied health care surveys) to ensure a short window of recollection. SB group data will also be collected at these age markers, though this group will not have had exposure to LIFEspan. For retrospective participants, once consent is received, the ABI/CP participants will be ‘followed’ for a three-year period preceding introduction of the LIFEspan model. This window will match the age-related collection periods outlined for the prospective participants (i.e., first and second baseline, and post-transfer).

**Table 2:** Timeline of the Study

**Protocol Events**	**Timeframe**		
	**Year 1**	**Year 2**	**Year 3**
	**Baseline 1**	**Baseline 2**	**Outcomes**
	**Age: 16-17**	**Age: 17-18**	**Age: 18-19**
	**1 Year Pre-Transition**	**Transition Year**	**1 Year Post-Transition**
***Recruitment and Consent***	☑		
***Demographic Questionnaire Form***	☑		
***MOHLTC Billing Summaries***	☑	☑	☑
▪ Medical Specialists			
▪ Family Doctor			
▪ Emergency Services & Hospitalizations			
***Chart Audit***	☑	☑	☑
▪ LIFEspan Service Delivery Description			
▪ Demographics, Functional Level, & Diagnoses			
▪ Medical Specialists			
▪ Hospital Allied Care			
***“Allied Care” and “Community Involvement” Forms***	☑	☑	☑
▪ Community Allied Care	+ 6-months follow-up	+ 6-months follow-up	+ 6-months follow-up
▪ Vocation, Recreation, Social Activities, & Sports			
***Self-Reported Secondary Measures***	☑	☑	☑
▪ Transition Readiness			
▪ Health Status & Well-Being			
▪ Participation			
***Youth and Parent Interviews***			☑
▪ Transition Preparation & Experience			

#### Quantitative data

LIFE-H, HUI3, AQoL, and Arc assessment tools have been designed for use with individuals with disabilities; and thus, have low reading levels and do not include abstract questions that might be challenging to adolescents [19]. PIH and Self-Efficacy questionnaires are being trialed for the first time with the populations of participants enrolled in the study. Clinicians working with these participant groups have reviewed the tools for potential issues in comprehension. In addition, it will be emphasized to the participants that unlike school tests, the questions on these surveys have no right or wrong answers. Considerations of special accommodations, such as the provision of additional time, convenient locations, and functional and/or communicative assistance, will also be made, in accordance with recommendations in the literature [58]. Despite the potential for bias, completion by proxy respondents (e.g., parents, caregivers) will be sought where participants are unable to complete the measures by means of self-report. In these circumstances, parents’ views may be equally valuable as they will continue to play a significant and active role in managing the young person’s health care transition.

The amount of time required for the data collection process is expected to range from 1 to 4 hours, depending on the participants’ needs and accommodations required for completing the assessments based on their level of functioning. Secondary mail-outs and follow-up phone calls are expected to be required in most cases. In the event of missing information or uncompleted questionnaires, follow-up phone calls will be made. As personal claims histories will be generated by the MOHLTC in hard-copy format, study personnel will code and manually enter this information along with all data collected during the annual and bi-annual participant sessions. Each participant will be assigned a unique identifier to anonymize their data and facilitate linkage of the multiple data sources within a master spreadsheet. Since all data will be manually entered, we will ensure the accuracy of data by entering numerical data from all questionnaires twice, and establishing a 90% inter-rater reliability on 20% of chart audit entries and MOHLTC data that require coding.

#### Qualitative data

During the semi-structured interviews, participants will be provided with a hand-held stop-and-go sign they can use when they feel comfortable going ahead with the next question or stopping when they do not wish to answer a question. The use of such images may help minimize the adult-child (or experimenter/participant) power differential and ensure that participants feel comfortable and in control at all stages of the interview. The interviews will be digitally recorded to permit verbatim transcription. For the first 4 to 6 interviews, research team members skilled in qualitative interview techniques will review the transcripts and modify the interview guide as required. This process will highlight questions that require repeated clarification or rewording; identify new questions that may need to be posed; and allow a refinement of the script that is in keeping with a more natural conversation. We will verbally inform these first few participants that we are piloting the interview to ensure that ‘we are asking the right questions in the right way’. They will also be informed that we may follow-up and ask if they would be willing to respond to a few additional questions if significant changes to the script are made. This would be done by phone; if participants verbally consent, their answers will be recorded and included with their original interview transcript.

### Power calculation

Based on Young et al.’s study [13], we expect that 25% of young adults transferring from pediatric to adult services will meet our continuity of care definition (i.e., at least one visit to a primary care provider during the first year post-transition). It is hypothesized that the LIFEspan model will increase continuity of care to 75% in the intervention group (i.e., a three-fold increase). A sample size of 88 youth for the prospective comparison (70 Intervention and 75% continuity of care versus 18 Comparison and 25% continuity of care) will provide 90% power to detect this difference at an alpha level of 0.05. A similar retrospective group will also be required. Given the large sample size, the comparison between the prospective and retrospective group of participants with ABI and CP will also have very high power to detect this clinically important difference.

### Data analyses

#### Quantitative analyses

The audit information collected about each visit will be entered into a statistical software package (SAS®) and will include categorical data (yes/no), frequency counts, and calculated percentages. SAS® software will be used to conduct a descriptive analysis and generate graphical representations, measures of central tendency, and calculations of confidence intervals. This analysis will compare actual practice with practices proposed in the LIFEspan model. A similar analysis will be conducted comparing practices in the LIFEspan model to the standard of care model. For the subset of variables that are subject to participant variability, further analyses using polytomous logistic regression will compare service provided by degree of disability and diagnosis to identify factors associated with greater or lesser than expected use of services predicted by LIFEspan. Because of the large number of comparisons this entails, the analysis will be limited to univariate models and will be exploratory in nature. Rather than reporting p-values as being greater or lesser than .05 or .01, we will report the exact p-values.

Using SAS® software, multiple regression (health, well-being, social participation, and transition readiness scales), logistic regression (continuity of care and community involvement) and Poisson regression (health care utilization) analyses will be conducted to determine if there are differences between the intervention and comparison groups after controlling for disability, severity, basic demographics (e.g., SES, gender), and baseline data on both the outcome of interest and other baseline measures, taking into account within subject correlations. Planned contrasts will be used to compare outcomes for the intervention group with the prospective SB group as well as comparing outcomes for the intervention group with the retrospective ABI/CP group. A within intervention comparison between LIFEspan ABI and LIFEspan CP will also be conducted.

#### Qualitative analyses

Transcribed data will be inputted into NVivo® software to support the qualitative data analysis. General guiding questions and a semi-structured design will support the emergence of categories and codes *a posteriori*. The analyses will begin by each investigator independently reading each transcript several times. Through inductive analysis and an iterative process of organizing the data, patterns and themes will be identified. Through group discussion and consensus, codes will be clustered by topic and connections identified between common themes to form super-ordinate themes. The transcripts will be subsequently re-examined and coded according to the thematic branches identified during these preliminary stages. Using a constant comparative approach with continual adjustment throughout the process, codes will be examined, compared, and merged, relabeled or split as necessary. Codes resulting from this open coding process will be reviewed with reference to structure and relations between them. Finally, an advanced level of coding will be conducted which aims to reorganize the data segments and assign those with similar meanings to a new category. Code-recode and peer examination will help establish the trustworthiness of theme identification and coding.

### Compensation

All participants will receive a monetary gift-card as a thank you for their enrolment and completion of baseline data measures, followed by additional gift-cards upon completion of each additional data collection period. This is an expectation of the research ethics committee, and recognizes the value of participants’ time. Participants who complete measures on-site will also be reimbursed for their traveling expenses and provided with a light meal, if desired. Similarly, mail costs will be absorbed to support this data collection strategy.

### Dissemination

The results of the study will be disseminated in academic publications and journal special issues, reports to youth and their families, and websites maintained by various disability and transition groups, as well as knowledge translation activities involving the LIFEspan team members. The LETS study project team included several knowledge users as investigators, whose interdisciplinary perspectives contributed to the design of the study from the outset and will serve to facilitate knowledge translation as the project rolls out and findings become available.

## Discussion

The LETS study will be unique in conducting a prospective examination of a recently funded coordinated long-term approach to care. Demonstration of outcomes experienced by LIFEspan clients will quantify the value of a coordinated transfer approach and provide data for future studies examining the long-term implications of such a model on continuity of care, quality of life, inclusion, and participation. In a recent paper, McDonagh and Kelly [19] attributed the current state of transition research in part to methodological challenges. Through a chronic illness lens, they identified key issues that have hindered development of a strong evidence base for the field of transition in solid organ transplantation, including a lack of consensus on basic terminology and adolescent age criteria, a lack of suitable measurement tools, the presence of heterogeneous financial and socio-cultural demographics, and in particular, the absence of guiding theoretical research frameworks to address “the complex multidimensional, multidisciplinary, and multiagency nature” (p. 690) of transition [19]. McDonagh and Kelly’s careful consideration of such challenges is validating for transition researchers attempting to make headway. Based on our experiences as a multidisciplinary team of pediatric and adult rehabilitation clinicians and researchers, we argue that these issues are not unique to transplantation but are common to the field of health care transition for youth with complex chronic conditions and disabilities broadly [27,28,59]. The current paper describes similar methodological challenges. We continue this discussion by providing concrete examples of such issues and the strategies used to address them as they were encountered in designing an active longitudinal study.

Despite these challenges, the LETS study has a number of methodological strengths. Unlike previous retrospective cross-sectional studies reported in the chronic condition and disability literature to-date [9,29-32,34,35], this prospective longitudinal study will examine the preparation for transition as well as their outcomes within one year post-transition. The outcome component of this study will determine whether LIFEspan establishes continuity of care within the first year post-transition, which is an important first step in maintaining long-term health. This window of time is previously unaccounted, as Young et al.’s study [14] examined a longer time frame (5 – 15 years) and did not provide information on the transition itself or its immediate consequences. Second, while there are no available measures of transition readiness, we will use a combination of measures that assess skills central to successful transition and thus reflect the multidimensional approach to transition, as recommended [19]. A wide range of measures with good psychometric properties and appropriate for individuals with disabilities will be implemented to assess outcomes such as quality of life, health, well-being, and emergency health care utilization. In addition, the qualitative component of the study will enrich this quantitative data and provide in-depth subjective perceptions of youth and their parents regarding their experiences of transition process, health care services, continuity of care, preparation interventions, etc. Finally, a comprehensive audit of implementation of LIFEspan (the process evaluation component) will allow for a detailed description of the intervention received by the participants. Previous evaluations described in the literature have not provided such detailed documentation of interventions.

Additionally, CP, ABI, and SB populations represent some of the most complex individuals who need support in transition and comprehensive, lifelong health and social services. Therefore, there is ample reason to believe that the findings from this study will generalize to a wide range of pediatric patients transitioning to adult systems. However, in general, heterogeneity is a huge issue in transition studies, due to the tremendous diversity across impairment groupings arising from variations in cognitive maturation, psychosocial development, skeletal and musculature growth, motor function and other co-morbidities [59]. Such variations manifest themselves in very different clinical presentations with implications for self-management, multi-disciplinary treatment needs, and multi-agency service utilization [59]. Conversely, exploring service needs and effective models of care within a single population is also extremely challenging given the vast number of potentially confounding factors associated with the potential spectrum of severity. While narrow inclusion criteria can be used to generate homogeneous samples and optimize data collection strategies, such efforts can diminish the representativeness of the sample by excluding participants who are more severely disabled, have worse health status, are least likely to achieve adequate societal participation, and who are, potentially, the most vulnerable with respect to lacking access to services that would respond to their complex needs. Thus, despite the demand for high level evidence, identification of an ethically sound and practical control group remains challenging within disability research and the field of transition [58]. In the current study, the issue of potential differences among population groups will be addressed by using multiple baselines (i.e., participants serving as their own controls) and including a retrospective control group from the same populations as those of the intervention group. Through its inclusive design, this study will offer a unique insight in the experiences of non-verbal participants via proxy reporting and inclusion of augmentative and alternative communication users, whose transition experiences to-date have not been incorporated in formal evaluations [60]. Studies supporting large samples and varied data collection techniques are still required as they will be critical to allow for natural variations in health and disability as well as socio-economic, geographical and cultural demographics among the youth.

Several limitations of this study should also be recognized. First, drop-out is always a significant risk in longitudinal designs. However, subject attrition for primary measures of continuity of care is expected to be minimal as permission for data release was sought at the outset of the study, and administrative database interrogation does not require continued participant contact. Unless a participant withdraws from the study, the primary outcome will be available on all participants, irrespective of loss to follow-up. In addition, key demographic data were also obtained at the time of consent; this information will allow for a detailed comparison of participants who completed the study with those that did not. However, subject attrition will still be concerning for secondary measures. Second, since this project is a longitudinal prospective cohort study of the LIFEspan model, the representativeness of the sample is also a potential risk. At present, the LIFEspan model is only available for clients of one pediatric centre. The centre is recognized as Canada’s largest rehabilitation centre and is located in a large multi-cultural urban area of Ontario. As such, LIFEspan does set the stage for an ideal model for which to strive in service delivery, where, for example, LIFEspan clients benefit from a publicly funded health care system and transportation services to and from the centre. However, generalizability of the LETS study is thereby somewhat limited, as these services and such multicultural populations might not be found in other jurisdictions. Third, the inclusion of three different disability groups also does raise the possibility of differences arising as a function of disability-related influences and not the intervention. While the standardized measures in this study have been selected based on their appropriateness for completion by people with disabilities, it is well recognized that individuals with physical and developmental disabilities may not follow the same developmental trajectory as their age-matched peers and as such, normative milestones may not be valid [61].

While there is a wealth of evidence articulating challenges to implementing effective models of care, empirical evidence addressing processes and outcomes of transition remains limited. The importance of continuing research on adolescent health care transitions is indisputable for both practice and policy development, since due to a multitude of factors related to systemic deficiencies and inadequate preparation of clients for transition to adulthood, adolescents with childhood-onset disability are at an increased risk of health problems, secondary disabilities, and failure to achieve optimal adult societal roles and community participation. Generally, the population of young adults with disabilities and complex chronic conditions and their families will benefit greatly from stronger partnerships between the pediatric and adult sector, consistent financing, and dedicated human resources [3]. However, there is a lack of written institutional guidance or policy regarding transition to influence such decisions, as existing research has not yet translated into policy [5,62]. Thus, systematic evaluations are essential to determine the success of transition programs and address this gap. The LETS study is unique in design, aims to address gaps in the literature, and will provide evidence to support the expansion of the LIFEspan model and its adaptability to other organizations, within different clinical settings, and for clients with other childhood disabilities. The current paper serves as an important step in the dissemination of the results by outlining the project background, explaining its key concepts, and providing a detailed description of methods adopted prior to the results of the analyses.

## Abbreviations

ABI: Acquired brain injury; CP: Cerebral palsy; LETS: Longitudinal Evaluation of Transition Services; LIFEspan: Living Independently and Fully Engaged Across the Lifespan; SB: Spina bifida; MOHLTC: Ministry of Health and Long-Term Care.

## Competing interests

The authors declare that they have no competing interests.

## Authors’ contributions

SK, JM, MB, CM, and HH conceptualized the study; SL, PM, AC, and YH contributed to the design of the study and were investigators/consultants on the grant application. IT participated in the coordination of the study. All authors participated in the drafting of this manuscript, and all authors read and approved the final manuscript.

## Pre-publication history

The pre-publication history for this paper can be accessed here:

http://www.biomedcentral.com/1471-2431/12/51/prepub
